# International multicenter real-world REGistry for patients with metastatic renAL cell carcinoma – Meet-URO 33 study (REGAL study)

**DOI:** 10.1186/s12885-024-12319-1

**Published:** 2024-06-24

**Authors:** Sara Elena Rebuzzi, Giuseppe Fornarini, Alessio Signori, Sebastiano Buti, Giuseppe Procopio, Ugo De Giorgi, Sandro Pignata, Emanuele Naglieri, Marco Maruzzo, Giuseppe Luigi Banna, Pasquale Rescigno, Carlo Messina, Alvise Mattana, Umberto Basso, Davide Bimbatti

**Affiliations:** 1 Medical Oncology Unit, Ospedale San Paolo, Savona, Italy; 2 Department of Internal Medicine and Medical Specialties (Di.M.I.), University of Genova, Genova, Italy; 3 Medical Oncology Unit 1, IRCCS Ospedale Policlinico San Martino, Genova, Italy; 4 Department of Health Sciences (DISSAL), Section of Biostatistics, University of Genova, Genova, Italy; 5 Medical Oncology Unit, University Hospital of Parma, Parma, Italy; 6 Department of Medicine and Surgery, University of Parma, Parma, Italy; 7 Medical Oncology, Fondazione IRCCS – Istituto Nazionale dei Tumori, Milano, Italy; 8 Medical Oncology Department, IRCCS Istituto Romagnolo per lo studio dei tumori (IRST) “Dino Amadori”, Meldola, Italy; 9 Department of Urology and Gynecology, Istituto Nazionale Tumori IRCCS Fondazione G. Pascale, Napoli, Italy; 10 U.O. Oncologia, IRCCS Istituto Tumori Giovanni Paolo II, Bari, Italy; 11 IOV - IRCCS, Oncologia 1, Istituto Oncologico Veneto, Padova, Italy; 12 Portsmouth Hospitals University NHS Trust, Portsmouth, PO6 3LY UK; 13 Faculty of Science and Health, School of Pharmacy and Biomedical Sciences, University of Portsmouth, Portsmouth, PO1 2UP UK; 14 Translational and Clinical Research Institute, Centre for Cancer, Newcastle University, Newcastle Upon Tyne, UK; 15 Candiolo Cancer Institute, FPO-IRCCS, Candiolo, Italy; 16 Oncology Unit, ARNAS Civico, Palermo, Italy

**Keywords:** Metastatic renal cell carcinoma, Immunotherapy, Immune checkpoint inhibitor, Clinical practice, Real-world, IMDC score, Meet-URO score, Prospective, Retrospective

## Abstract

**Background:**

Nowadays, different therapeutic options are available for the first-line treatment of metastatic renal cell carcinoma (mRCC). Immuno-combinations are the standard first-line therapy in all mRCC patients regardless of the International Metastatic RCC Database Consortium (IMDC) risk category, even though TKI monotherapy is still a therapeutic option in selected patients. However, comparisons between the different first-line treatment strategies are lacking and few real-world data are available in this setting. For this reason, the regimen choice represents an important issue in clinical practice and the optimal treatment sequence remains unclear.

**Methods:**

The REGAL study is a multicentric prospective observational study enrolling mRCC patients treated with first-line systemic therapy according to clinical practice in a real-world setting. A retrospective cohort of mRCC patients who received first-line systemic therapy from the 1st of January 2021 will also be included. The primary objective is to identify potential prognostic and predictive factors that could help guide the treatment choice; secondary objectives included the assessment of the prognostic performance of the novel prognostic Meet-URO score (IMDC score + neutrophil-to-lymphocyte ratio + bone metastases) compared with the IMDC score and the comparison between treatment strategies according to response and survival outcomes and toxicity profile.

**Discussion:**

Considering the high number of therapeutic first-line strategies available for mRCC, the identification of clinical prognostic and predictive factors to candidate patients to a preferable systemic therapy is still an unmet clinical need. The Meet-URO 33 study aims to provide a large-scale real-world database on mRCC patients, to identify the clinical predictive and prognostic factors and the different performances between the ICI-based combinations according to response, survival and toxicity.

**Trial Registration:**

CESC IOV 2023-78.

## Background

For decades, the standard treatment of mRCC was based on the inhibition of angiogenesis using the vascular endothelial growth factor receptor tyrosine kinase inhibitors (VEGFR-TKIs) [1, 2]. Over the past 5 years, immune-checkpoint inhibitors (ICIs) have significantly improved the therapeutic landscape of mRCC [3, 4]. In 2015, nivolumab was the first ICI approved for the treatment of mRCC patients progressing to VEGFR-TKIs [5, 6].

More recently, ICI-based combinations have become the novel first-line standard of care, according to their superiority in terms of response and survival outcomes compared with sunitinib [7–12]. Based on the results of phase III trials, four combination strategies are now available in Europe for the treatment of mRCC: nivolumab plus ipilimumab for IMDC intermediate- and poor-risk patients and pembrolizumab plus axitinib, nivolumab plus cabozantinib and pembrolizumab plus lenvatinib regardless of the IMDC risk group.

However, the lack of direct comparisons between these ICI-based combinations represents an urgent issue in clinical practice. Nowadays, the regimen choice is based on the toxicity profile, the need for tumor shrinkage and disease control which seems to be better reached with TKI + ICIs combinations, and on the presence of sarcomatoid features, on which TKIs seem to be less effective [13, 14].

Furthermore, in favorable risk classes, ICIs + TKIs combinations showed a benefit in progression-free survival (PFS) but not in overall survival (OS), suggesting that some selected categories of patients with indolent disease could still benefit from the TKI monotherapy [15, 16].

Given the availability of ICI-based combinations in the first-line, establishing the optimal therapeutic sequence remains undefined [17]. Cabozantinib monotherapy is the recommended option after failure of immune combinations given its multitarget activity and the efficacy reported by retrospective data [18].

According to the different first-line combination strategies available and the lack of any direct comparison between these regimens, the identification of clinical and biological prognostic and predictive factors to select patients and help the clinicians’ therapeutic choice is still an unmet clinical need. For this reason, a new prognostic score (Meet-URO score) was developed from a multicentric retrospective Italian study on 571 mRCC patients receiving 2nd line nivolumab [19]. The Meet-URO score combines the IMDC score with two well-known risk factors, the presence of bone metastases and the neutrophil-to-lymphocyte ratio (NLR). The Meet-URO score demonstrated a higher prognostic accuracy compared with the IMDC score alone, identifying 5 risk groups with different survival outcomes [19].

The aim of the Meet-URO 33 study (REGAL study) is to set up a large-scale real-world database to identify prognostic and predictive factors to help clinical decision-making, compare the Meet-URO score and the IMDC score, and assess the different response and survival outcomes and toxicity profile of the different immuno-combinations.

## Methods/design

### Study design

The Meet-URO 33 study (REGAL study) is an international multicentric prospective observational study, which includes patients with a histological diagnosis of RCC and advanced stage treated with first-line systemic therapy in a real-world setting. A retrospective cohort of mRCC patients who underwent first-line systemic therapy from the 1st of January 2021 will also be included.

Eighty-four Italian centers are included in the study and a study amendment will be submitted to include about 10 European centers.

For its registry nature, this study does not have a maximum limit of patients analyzed. Overall, it is estimated to enroll approximately 200 patients in the retrospective part and over 500 patients in the prospective part. For the prospective part, the enrollment will last about 5 years.

### Recruitment method

Before performing any study procedure, the signature of informed consent is required from each patient. A copy of the consent will be given to the patient. After confirming the presence of all the inclusion criteria and the absence of all exclusion criteria, the patient can be enrolled in the observational study.

### Eligibility criteria

#### Inclusion criteria

Participants meeting all of the following criteria will be considered for enrollment:

- Age ≥ 18 years old;
- Histological diagnosis of RCC and advanced stage treated with first-line systemic therapy according to clinical practice, which was started from the 1st of January 2021;
- Availability of complete oncological and medical records;
- Written informed consent signed.

#### Exclusion criteria

Participants meeting one of the following criteria will be excluded from enrollment in this study:

- Histological diagnosis of non-RCC (e.g. urothelial carcinoma, sarcoma);
- mRCC patients in active surveillance;
- No clinical data available.

### Treatment

In consideration of the non-interventional, descriptive and observational nature, the present study will not influence the current or future therapeutic choices (immuno-combinations or TKI monotherapies). Patients will be treated with different systemic and/or local therapies, as part of normal clinical practice, regardless of study enrollment.

### Objectives

#### Primary objective

The primary objective of the study is the identification of potential prognostic and/or predictive factors of mRCC. Details on potential prognostic factors are detailed below in the data collection chapter.

#### Secondary objectives

The secondary objectives included:

- to compare different first-line and subsequent-line oncological treatments according to response and survival outcomes and toxicity profile. Endpoints investigated will be objective response rate (ORR), disease control rate (DCR), progression-free survival (PFS) and overall survival (OS);
- to assess the correlation between the clinical and tumor characteristics and the choice of the first line of treatment;
- To assess the prognostic accuracy of the Meet-URO score compared with the standard IMDC score.

#### Exploratory objectives

Given the observational and prospective nature of the study, further studies will be planned subsequently, both on the entire cohort and particular subgroups (e.g. poor-risk category, elderly, non-clear cell histology).

### Data collection

An international database of mRCC patients undergoing first-line systemic therapy will be established on the RedCap platform. In particular, the subsequent data will be required: name of the center, patient code, sex, date of birth, histology, grading and molecular assessments if available, date of the first diagnosis, date of the metastatic disease diagnosis, data on surgery or locoregional treatments for localized disease if performed, sites of metastatic disease, starting and ending date for each systemic treatment, surgical or locoregional procedures performed for metastatic disease, blood chemistry performed before and during treatment, Eastern Cooperative Oncology Group Performance Status (ECOG PS) before starting systemic therapy, toxicity profile (focusing on G3-G4 adverse events), best radiological response, site and date of progressive disease, last follow-up date, death.

### Statistical analysis

The descriptive statistics will be used to summarize the clinical characteristics of patients and the distribution of possible prognostic factors. All time-to-event endpoints (PFS, OS) will be analyzed using the Kaplan-Meier method, the restricted mean survival time (RMST) and the Cox proportional hazard regression model. PFS and OS will be calculated from the date of treatment start up to the date of the event or the last follow-up for censored patients.

The binary endpoints (ORR, DCR) will instead be analyzed through relative frequencies and logistic regression.

For all the comparisons between treatments, all causal inference techniques such as propensity scores and marginal structural models will be used. All the steps for a correct target trial emulation strategy will be followed to avoid potential biases deriving from the observational nature of the study.

In particular, in comparing the different treatments, the principles of emulating a clinical trial will be applied [20], developing appropriate ad hoc protocols for each planned comparison.

## Discussion

The treatment landscape of mRCC patients has been revolutionized with the onset of different first-line immuno-combinations in the last few years. No head-to-head comparison data are available and no predictive biomarkers have been identified. Due to these limits, the choice between the different therapeutic regimens is now guided by clinical and tolerability parameters, on regulatory approval and the possible treatment sequence.

Recently, several reviews and meta-analyses have been performed comparing the multiple first-line treatments for mRCC [7, 14, 21–23]. One of the most recent ones [14] reported that pembrolizumab plus lenvatinib was associated with the highest probability of providing PFS and OS benefit and yielded the highest probability of being the best treatment in terms of PFS regardless of the IMDC risk group. Moreover, in this meta-analysis nivolumab plus cabozantinib and nivolumab plus ipilimumab had the highest possibility to be the best treatment for the sarcomatoid subgroup. However, these data as the ones from the previous meta-analyses and reviews, may be based on partial and immature data and should be taken with caution since formal comparisons are still urgently awaited.

With the institution of a large-scale real-world database on mRCC patients starting first-line therapy, head-to-head comparisons will be performed between the different immune combinations according to response and survival outcomes and toxicity profile. This project is fundamental to identifying the more effective treatment according to clinical and tumoral prognostic and predictive factors. Among them, the Meet-URO score will be assessed in this context and compared with the IMDC score.

Moreover, the study will also analyze the different toxicity profiles in a real-world population, providing more data to help clinicians in decision-making. The implementation of the study with ad-hoc sub-analysis on specific patients or tumor subgroups will also be encouraged.

Among the potential limitations of this study, we acknowledge the presence of the retrospective cohort with the subsequent lack of all clinical data and the absence of an independent radiological review of the imaging for the disease reassessments due to the observational nature of the study.

In conclusion, this study aims to provide new real-world evidence on the first-line and subsequent therapies in mRCC and answers to the issues still present in clinical practice.

## Data Availability

The datasets used and/or analyzed during the current study will be available from the corresponding author upon reasonable request.

## Abbreviations

mRCC: Metastatic Renal Cell-Carcinoma
IMDC: International Metastatic RCC Database Consortium Risk Model for Metastatic Renal Cell Carcinoma
TKIs: Tyrosine-kinase Inhibitors
VEGFR-TKI: Vascular Endothelial Growth Factor Receptor Tyrosine Kinase Inhibitor
ICIs: Immune-checkpoints Inhibitors
PFS: Progression-free Survival
OS: Overall Survival
NLR: Neutrophil-to-Lymphocyte Ratio
ECOG PS: Eastern Cooperative Oncology Group Performance Status
KM: Kaplan-Meier method
RMST: Restricted Mean Survival Time
ORR: Overall Response Rate
DCR: Disease Control Rate
